# Caffeic Acid Phenethyl Ester as a Potential Treatment for Advanced Prostate Cancer Targeting Akt Signaling

**DOI:** 10.3390/ijms14035264

**Published:** 2013-03-06

**Authors:** Hui-Ping Lin, Ching-Yu Lin, Chun-Chieh Liu, Liang-Cheng Su, Chieh Huo, Ying-Yu Kuo, Jen-Chih Tseng, Jong-Ming Hsu, Chi-Kuan Chen, Chih-Pin Chuu

**Affiliations:** 1 Institute of Cellular and System Medicine, National Health Research Institutes, Miaoli 35053, Taiwan; E-Mails: diablofish@nhri.org.tw (H.-P.L.); cylin071@gmail.com (C.-Y.L.); liangcheng610@nhri.org.tw (L.-C.S.); jason429w@yahoo.com.tw (C.H.); jennykuo0101@yahoo.com.tw (Y.-Y.K.); s100080826@m100.nthu.edu.tw (J.-C.T.); 2 Translational Center for Glandular Malignancies, National Health Research Institutes, Miaoli 35053, Taiwan; 3 National Institute of Cancer Research, National Health Research Institutes, Miaoli 35053, Taiwan; 4 Division of Cardiology, Department of Internal Medicine, Mackay Memorial Hospital, Taipei City 10449, Taiwan; E-Mail: jasonliu0528@gmail.com; 5 Mackay Medical College, New Taipei City 25245, Taiwan; 6 Mackay Medicine, Nursing and Management College, New Taipei City 25245, Taiwan; 7 Department of Life Sciences, National Central University, Taoyuan City 32001, Taiwan; 8 Institute of Molecular and Cellular Biology, National Tsing Hua University, Hsinchu City 30013, Taiwan; 9 Department of Urology, Mackay Memorial Hospital, Taipei City 10449, Taiwan; E-Mail: jmhsu7@yahoo.com.tw; 10 Department of Pathology, Mackay Memorial Hospital, Taipei City 10449, Taiwan; E-Mail: chenchikuan@googlemail.com; 11 Graduate Program for Aging, China Medical University, Taichung City 40402, Taiwan; 12 Ph.D. Program in Tissue Engineering and Regenerative Medicine, National Chung Hsing University, Taichung City 40227, Taiwan

**Keywords:** prostate cancer, caffeic acid phenethyl ester, Akt, LNCaP, PC-3

## Abstract

Prostate cancer is the fifth most common cancer overall in the world. Androgen ablation therapy is the primary treatment for metastatic prostate cancer. However, most prostate cancer patients receiving the androgen ablation therapy ultimately develop recurrent castration-resistant tumors within 1–3 years after treatment. The median overall survival time is 1–2 years after tumor relapse. Chemotherapy shows little effect on prolonging survival for patients with metastatic hormone-refractory prostate cancer. More than 80% of prostate tumors acquire mutation or deletion of tumor suppressor phosphatase and tensin homolog (PTEN), a negative regulator of PI3K/Akt signaling, indicating that inhibition of PI3K/Akt might be a potential therapy for advanced prostate tumors. Caffeic acid phenethyl ester (CAPE) is a strong antioxidant extracted from honeybee hive propolis. CAPE is a well-known NF-κB inhibitor. CAPE has been used in folk medicine as a potent anti-inflammatory agent. Recent studies indicate that CAPE treatment suppresses tumor growth and Akt signaling in human prostate cancer cells. We discuss the potential of using CAPE as a treatment for patients with advanced prostate cancer targeting Akt signaling pathway in this review article.

## 1. Introduction

Prostate is a gland in the male reproductive system. It secretes a milky or white slightly acidic fluid constituting 50%–75% of semen along with spermatozoa and seminal vesicle fluid. Prostate cancer is the cancer develops in the prostate. Prostate cancer is the second most frequently diagnosed cancer of men and the fifth most common cancer overall in the world. The majority of patients having prostate cancer are over 65 years old and the 5 years survival rate for prostate cancer patients is more than 80%. Nearly 900,000 new cases have been diagnosed in 2008 (GLOBOCAN 2008 database, version 1.2). According to the statistics of Surveillance Epidemiology and End Results (SEER) of National Cancer Institute, more than 24,000 men have been diagnosed with and more than 28,000 men have died of prostate cancer in 2012 in United States. Many factors, including genetics and diet, have been implicated in the development of prostate cancer [1]. Examinations of prostate cancer include physical examination and serum prostate-specific antigen (PSA) test. PSA, also known as kallikrein-3 (KLK3), is a glycoprotein enzyme encoded in humans by the KLK3 gene. PSA level elevates when prostate develop cancer or other diseases [2,3]. PSA is a target gene of androgen receptor (AR) [4,5]. AR plays essential roles in the development of male sex organs and prostate tissues. AR also plays important roles in the development, progression, and metastasis of prostate cancer [6–11]. In prostate cancer cells, AR modulates the expression of proteins regulating cell cycle, survival, and growth [8–14]. AR stimulates the expression of TMPRSS2: ERG, a common gene fusion associated with prostate cancer [15–17]. Elevation of AR mRNA and protein expression has been observed in hormone-refractory prostate tumors compared to the primary androgen-dependent prostate tumors [18–26]. Amplification of the AR locus is reported in nearly one-third of patients developing hormone-refractory prostate cancers [20,23,27–29].

Surgery is often successful for organ-confined prostate cancer. Approximately 20%–40% of patients being treated with radical prostatectomy have tumor recurrence and elevation of serum prostate-specific antigen (PSA) [30]. More than 80% of prostate cancer patients die from bone metastases [31–33]. Bones and lymph nodes are the most common metastatic sites for prostate cancer, and the bone metastases cause severe pain. In 1941, Dr. Charles Huggins discovered that deprivation of androgen caused regression of hormone-responsive metastatic prostate cancer [34]. Since then, androgen ablation therapy has become the primary treatment for metastatic prostate cancer. Current androgen ablation therapy uses luteinizing hormone-releasing hormone agonists (LH-RH) (also known as gonadotropin-releasing hormone, GnRH) [35]. However, the majority of prostate cancer patients receiving androgen ablation therapy develop recurrent castration-resistant tumors within 1–3 years after treatment. The median overall survival time is 1–2 years after cancer relapse [8,36]. Chemotherapy is often used to treat metastatic hormone-refractory prostate cancer [37]. Commonly used chemotherapeutic drugs for prostate cancers include etoposide, paclitaxel, vinblastine, mitoxantrone, and estramustine. Etoposide and mitoxantrone are type II topoisomerase inhibitor [37,38]. Estramustine is a derivative of estrogen with a nitrogen mustard-carbamate ester moiety [37]. Vinblastine binds tubulin and inhibits assembly of microtubules [37]. Paclitaxel disrupts mitotic spindle assembly, chromosome segregation, and cell division. Paclitaxel also stabilizes the microtubule polymer and protects it from disassembly [37]. Treatments with these chemotherapeutic drugs result in decrease of PSA, radiographic response, and improvement of pain and urinary symptoms in a sub-group of patients. However, chemotherapies show little effect on prolonging survival [37]. Undesired side effects of these chemotherapeutic agents include toxic deaths, strokes, thrombosis, neutropenia, edema, dyspnea, malaise, and fatigue [37]. Therefore, alternative treatments for advanced prostate cancers are needed. The aim of this review article is to investigate the possibility of using caffeic acid phenethyl ester (CAPE), a pure natural compound extracted from honeybee hive propolis, for treatment of patients with advanced prostate cancer via inhibition of Akt signaling.

## 2. Akt Signaling and Prostate Cancer

Akt is a serine/threonine protein kinase regulating a variety of cellular responses, including inhibition of apoptosis and stimulation of cell proliferation. There are three mammalian isoforms of this enzyme, Akt1, Akt2, and Akt3 [39,40]. Phosphatase and tensin homolog (PTEN) protein acts as a phosphatase to dephosphorylate phosphatidylinositol (3,4,5)-trisphosphate. PTEN negatively controls the phosphoinositide 3-kinase/Akt signaling pathway [41]. PTEN is one of the most commonly mutated tumor suppressor genes in human cancers. PTEN is frequently deleted or mutated in prostatic intraepithelial neoplasia (PIN) and prostate cancer, resulting in activation of PI3K/Akt signaling [42,43]. Proteins of the ETS family are group of transcription factors regulating cell proliferation, differentiation, and carcinogenesis. In prostate cancer, fusion of ETS-related genes (in most cases, the ERG) with AR-regulated gene promoter of TMPRSS2 is present in approximately 50% of prostate tumors [44]. Recent studies indicate that gene fusion of TMPRSS2-ERG promotes prostate cancer when PTEN is concurrently lost [44–46]. There are two phosphorylation sites on Akt, threonine 308 and serine 473. Phosphorylation of Thr308 on Akt is activated by PDK1 [47]. Phosphorylation of serine 473 is activated by mTOR kinase, its associated protein rector, and SIN1/MIP1 [48,49].

PI3K/Akt signaling plays important roles in survival and progression of prostate cancer cells [42]. Immunoreactivity assay indicates that level of phospho-Akt correlates with higher Gleason score and immunoreactivity for Ki67 and phospho-epidermal growth factor receptor (EGFR) [50]. EGFR is a member of the ErbB family of receptor tyrosine kinase (RTK) which plays essential role in regulating cell proliferation and signaling transduction [51,52]. Expression levels of phospho-Akt and phospho-mTOR correlate with Gleason score [53]. Up-regulation of PI3K/Akt activity is associated with poor clinical outcome of prostate cancer [43,54–59]. Growth factor signaling pathways have been reported to cross-talk with AR-signaling in prostate cancer cells. Growth factor signaling pathways promote androgen-independent proliferation of prostate cancer cells and activate AR by modifying the phosphorylation status of AR or by altering the expression of coactivators or inhibitors [57,60]. The cross-talks between AR and growth factor signaling pathways sensitize AR to suboptimal level of androgenic stimulation [57,60]. AR transcriptional activity and expression have been reported to be regulated by Akt [61]. Androgens regulate Akt pathway by both genomic and non-genomic effects [61]. Different modes of interaction between the AR and Akt pathways include direct interaction or regulation via downstream Wnt/GSK-3β/β-catenin pathway, NF-κB, and FOXO family members [61]. Activation of PI3 kinase/Akt pathway enhances AR activation in response to low level of androgens [62,63]. Therefore, small molecule inhibitors that can suppress PI3K/Akt signaling with minimal side effects are potential candidates for prostate cancer treatment. There are currently a few drugs targeting Akt signaling under clinical trials for prostate cancer treatments, including agents such as celecoxib, perifosine and genistein [64].

## 3. Caffeic Acid Phenethyl Ester (CAPE)

**Figure 1:**
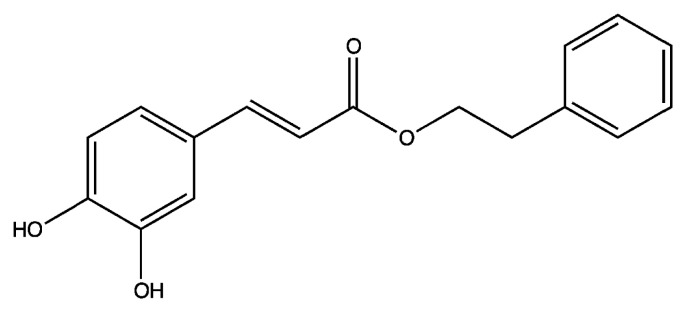
Structure of caffeic acid phenethyl ester (CAPE).

Caffeic acid phenethyl ester (CAPE) (Figure 1), a bioactive component extracted from honeybee hive propolis, is a strong antioxidant [65,66]. CAPE is a lipophilic derivative of caffeic acid and a phenolic antioxidant structurally related to 3,4-dihydroxycinnamic acid. CAPE has been used in folk medicine as a potent anti-inflammatory agent and is known to exhibit anti-mitogenic, anti-carcinogenic, anti-inflammatory, anti-viral, and immuno-modulatory properties. CAPE is a well-known NF-κB inhibitor. CAPE treatment at concentrations of 50 μM to 80 μM inhibits the activation of NF-κB via preventing the translocation of p65 unit of NF-κB [66] and preventing the binding between NF-κB and DNA [66].

## 4. Anti-Cancer Effects of CAPE on Human Cancer Cell Lines

**Figure 2:**
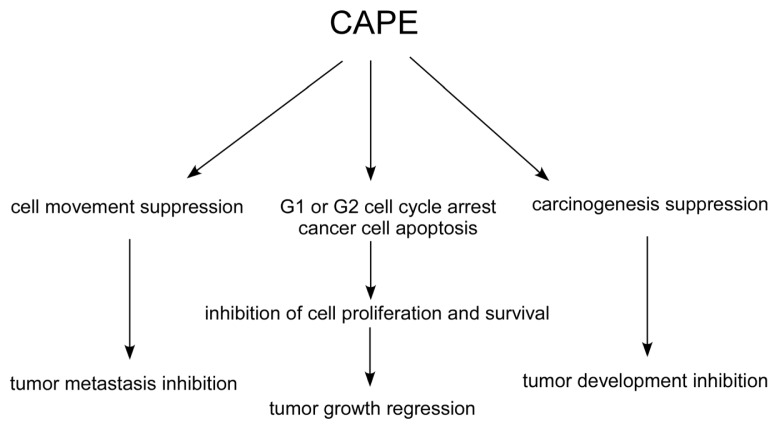
Effects of CAPE treatment on human cancer cells. Treating human cancer cells with CAPE causes suppression of carcinogenesis, cell cycle progression, and cell movement. CAPE also stimulates apoptosis in cancer cells. These changes then give rise to the inhibition of development, growth, and metastasis of tumors.

CAPE treatment inhibited the transformation of normal cells to cancer cells [67] (Figure 2). CAPE treatment suppressed proliferation of several human cancer cell lines, such as breast [68,69], prostate [70–72], lung [73,74], head and neck [75], cholangio [76], and cervical [77] cancer cells. Non-cancer human cells were much more resistant to CAPE treatment, indicating potential selective cytotoxic effect against cancer cells of CAPE treatment [73,75,78].

CAPE treatment caused apoptosis in cancer cells (Figure 2) via stimulation of Bax [69,79–81], Bak [81], p53 [67,77,81], p21^cip^[77], extracellular signal-regulated kinase (ERKs) [81], c-Jun [77], c-Jun *N*-terminal kinase (JNK) [69], p38 mitogen-activated protein kinase (p38 MAPK) [69,81], Fas ligand [69], caspase activity [77,79–81]. CAPE treatment decreased expression of Bcl-2 [80,82], cIAP-1, cIAP-2, and XIAP [72,79]. CAPE treatment also reduced release of cytochrome C [80,81] and loss of mitochondrial transmembrane potential [77].

In addition, CAPE treatment caused G1 or G2 cell cycle arrest in several cancer cells (Figure 2) through suppression of protein level of cyclin B1 [73,83], cyclin D1 [70,84,85], cyclin E [85], c-Myc [70,84], S-phase kinase-associated protein 2 (Skp2) [70], phospho-Rb [70,85], and β-catenin [86,87]. CAPE treatment increased protein expression of cyclin dependent kinase inhibitors p21^waf1/cip1^[70,85], p27^Kip1^[70,85], and p16^INK4A^[85].

CAPE treatment hindered motility and invasiveness of cancer cells (Figure 2) via suppression of Akt activity [88], focal adhesion kinase (FAK) activity [89], expression of matrix metalloproteinase MMP-2 and MMP-9, as well as disrupts the arrangement of actin cytoskeleton [89].

## 5. CAPE Suppresses Tumor Growth and Cancer Metastasis in Animal Models

Oral administration and intraperitoneal (i.p.) injection of CAPE prevented development of colon cancer [90–94] and liver cancer in the mice and the rat models. Intraperitoneal (i.p.) injection of CAPE suppressed tumor growth of melanoma and cholangiocarcinoma xenograft in the mice model [76,95]. Oral administration and i.p. injection of CAPE suppressed metastasis of colon cancer [91,96] and breast cancer [97] in the mice model. Subcutaneous and oral administration of CAPE suppressed liver metastasis of human HepG2 xenografts in the mice model [98].

## 6. Anticancer Effects of CAPE on Human Prostate Cancer Cells

LNCaP, PC-3, and DU-145 are the most commonly used cell lines for prostate cancer research. LNCaP, PC-3, and DU-145 cell lines are derived from human lymph node, bone, and brain metastatic lesion of prostate adenocarcinoma, respectively [99–102]. LNCaP cells express AR and PSA, while PC-3 and DU-145 cells do not express AR or PSA (Table 1).

**Figure 3:**
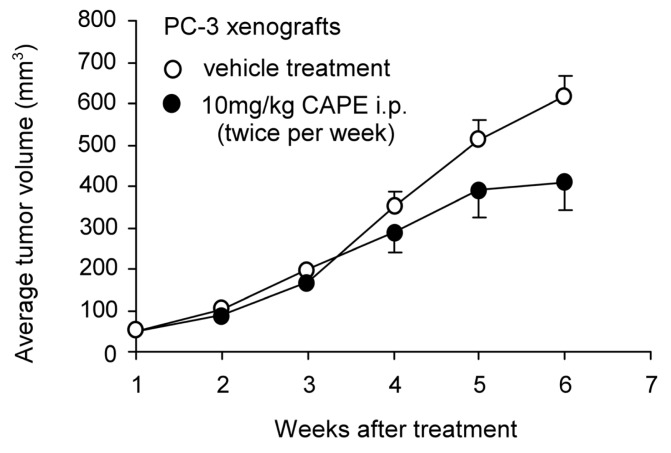
Effects of CAPE treatment on tumor growth of PC-3 xenografts in nude mice. Experiments involving mice were approved by the Institutional Animal Care and Use Committee of National Health Research Institutes. 6–8 week old male Balb/c nu/nu mice were injected subcutaneously in both flanks with 1 × 10^6^ PC-3 cells suspended in 0.5 mL of Matrigel (BD Bioscience, Franklin Lakes, NJ, USA). Tumors were allowed to grow for two weeks until the average tumor volume reached approximately 150 mm^3^. CAPE (10 mg/kg dissolved in DMSO and diluted in 0.1 mL PBS) or vehicle (DMSO with PBS) was then administered to mice by intraperitoneal injection (i.p.) twice a week for 5 weeks. Tumors were measured weekly using the formula: volume = length × width × height × 0.52. The CAPE treatment group comprised five mice with nine tumors while the vehicle control group comprised five mice with eight tumors. Tumor volume was shown as volume plus standard error (SE).

Our previous studies suggested that the proliferation of LNCaP 104-S, DU-145, and PC-3 human prostate cancer cells was dosage-dependently suppressed by CAPE treatment (IC_50_ 0.68 μM, 9.54 μM, and 18.65 μM, respectively) [70,71]. LNCaP 104-S is an androgen-dependent subline isolated from LNCaP FGC which is very sensitive to androgen treatment [103–105]. The growth inhibitory effects of CAPE treatment on LNCaP 104-S and PC-3 cells happened within 24 h following CAPE treatment and accumulated over time [70,71]. Treatment with 10 μM CAPE significantly inhibited the formation of LNCaP 104-S and PC-3 colonies in soft agar [70,71]. Flow cytometric analysis revealed that treatment with 3 μM to 20 μM CAPE reduced S phase cell population and caused G1 cell cycle arrest in LNCaP 104-S and PC-3 cells [70,71]. CAPE treatment at high concentrations (88 μM to 176 μM) induced apoptosis in PC-3 cells [72]. Administration of CAPE by gavage (10 mg/kg body weight per day) for six weeks resulted in 50% reduction of LNCaP 104-S xenografts tumor volume in nude mice (*p* = 0.0008) [70]. Intraperitoneal injection (i.p.) of CAPE (10 mg/kg body weight per day) twice a week for 5 weeks reduced 33% of PC-3 xenografts tumor volume in nude mice (*p* < 0.05, unpublished data, Figure 3).

The enzyme steroid 5α-reductase is responsible for the conversion of testosterone to 5α-dihydrotestosterone (DHT). In men, approximately 5% of testosterone undergoes 5α-reduction to form the more potent androgen, the DHT. 5α-reductase synthesizes DHT in the prostate, testes, hair follicles, and adrenal glands. Therefore, 5α-reductase activity is critical for male sexual differentiation and may be involved in the development of benign prostatic hyperplasia, alopecia, hirsutism, and prostate cancer [106]. CAPE treatment suppresses activity of type 1 and type 2 of 5α-reductase (IC_50_ 8 μM and 7 μM, respectively) [106]. Co-treatment of low concentrations (2.5 μM to 20 μM) of CAPE with chemotherapeutic drugs vinblastine, paclitaxol, or estramustine indicated synergistic suppression effect [71]. These observations suggested that CAPE administration may be useful for the treatment of prostate cancer and androgen-dependent disorders.

## 7. CAPE Inhibits Akt Signaling in Prostate Cancer Cells

**Figure 4:**
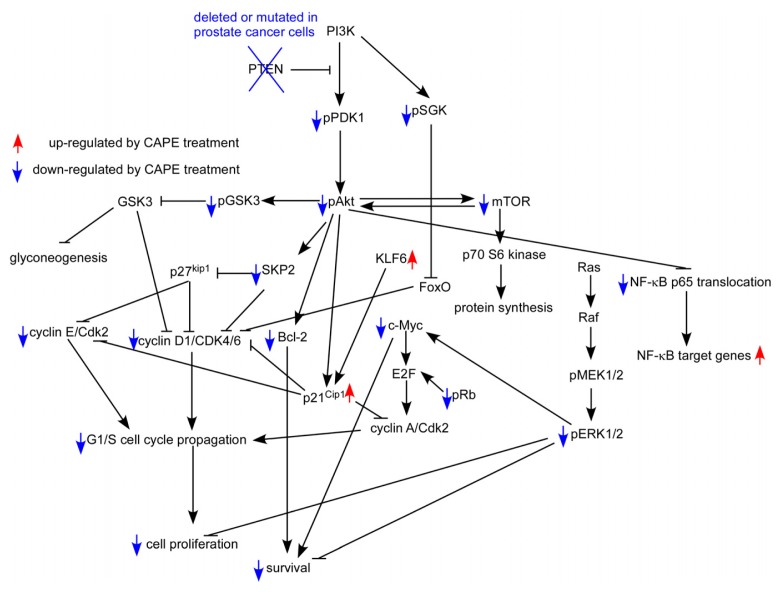
Putative model of anti-cancer effect of CAPE in human prostate cancer cells. Protein abundance or activity being stimulated by CAPE treatment are labeled with red upward arrows, while those being suppressed by CAPE treatment are labeled with blue downward arrows.

Although CAPE is a well-known NF-κB specific inhibitor, it does not affect NF-κB activity in LNCaP 104-S or PC-3 cells at concentration lower than 20 μM [70–72]. CAPE has previously been shown to suppress Akt signaling in human T cells, coronary smooth muscle cells, and lung cancer cells [88,107,108]. The PTEN in LNCaP cells is mutated, while PC-3 cells acquire a homozygous deletion of PTEN. Therefore, Akt is constantly active in these two cells. Reduction of PDK1 and mTOR activity contribute to the decrease of phosphorylation on Akt. The activities of GSK3α and GSK3β are inhibited by Akt-mediated phosphorylation at Ser21 and Ser9 respectively, limiting their ability to phosphorylate cell cycle regulating proteins, such as cyclin D1 and p21^Cip1^[109,110]. GSK3β-dependent phosphorylation of cyclin D1 mediates nuclear export and rapid degradation within the cytoplasm of cyclin D1 [111]. Decrease of phosphorylation of GSK3α and GSK3β due to decreased phosphorylation of Akt promotes elevated activity of GSK3α and GSK3β Upregulation of GSK3β activity decreases the abundance of cyclin D1. SGK, like Akt, promotes proliferation through phosphorylation-mediated inactivation of the forkhead transcription factor FoxO3 [112]. Skp2, a member of the F-box protein family, is responsible for ubiquitination and down-regulation of p27^Kip^ cell cycle inhibitors. Skp2 promotes cell cycle progression from G1 phase to S phase. Phosphorylation of Cdk2 on T160 is necessary for its activation [113] and is required for traversing the G1/S checkpoint through phosphorylation of Rb. Phosphorylation of c-Raf on S259 and S621 creates 14-3-3 binding sites which are thought to maintain it in an auto-inhibited state [114]. Treatment of 10 μM CAPE significantly decreased protein abundance of signaling protein involved in Akt signaling and cell cycle regulation, including Akt1, Akt2, Akt3, cyclin A, cyclin D1, cyclin E, SKP2, c-Myc, mTOR, Rb, Bcl-2, phospho-Akt Ser473, phospho-Akt Thr308, phospho-glycogen synthase kinase 3 alpha (Gsk3α Ser21, phospho-Gsk3β Ser9, phospho-mTOR Ser2448, phospho-mTOR Ser2481, phospho-PDK1 Ser241, phospho-Cdk2 Thr160, Ser259, and phospho-serum and glucocorticoid-inducible kinase (SGK) Ser78 in LNCaP 104-S and PC-3 cells [70,71]. CAPE treatment stimulated the protein expression of cell cycle inhibitor p21^Cip1^ and p27^Kip1^ as well as phospho-p38 MAPK Thr180/Tyr182 and phospho-p90RSK Ser380 [70,71]. Consistent with inactivation of Cdk2, phospho-Rb Ser807/811 was decreased by 70% under CAPE treatment. CAPE treatment led to increased levels of phospho-c-Raf Ser259. Down-regulation of cyclin A, c-Myc, Skp2, phospho-Rb, and Cdk2, coupled with increased phospho-c-Raf Ser259, p21^Cip^ and p27^Kip1^ abundance likely contributed to the sustained induction of G1 cell cycle arrest following CAPE treatment in LNCaP 104-S and PC-3 prostate cancer cells (Figure 4).

**Figure 5:**
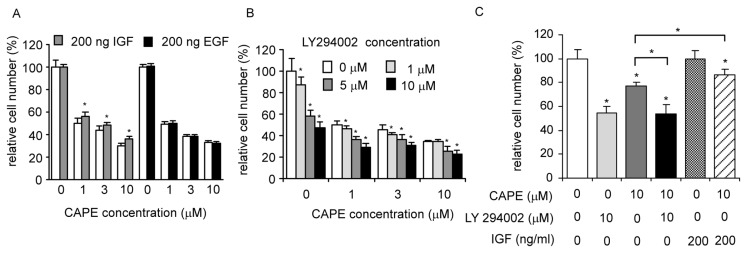
CAPE treatment targets PI3K/Akt signaling pathway. (**A**) LNCaP 104-S cells were treated with 0, 1, 3, or 10 μM CAPE in the presence and absence of 200 ng IGF-1 or 200 ng EGF for 96 h; (**B**) LNCaP 104-S cells was treated with CAPE (0, 1, 3, or 10 μM) and LY294002 (0, 1, 5, or 10 μM) for 96 h; (**C**) PC-3 cells was treated with different combinations of CAPE (10 μM), LY294002 (10 μM), and IGF-1 (200 ng/mL) for 72 h. Cell number was determined by 96-well proliferation assay. (*) represents statistically significant difference (*p* < 0.05) between the groups treated with IGF-1 or LY294002 compared to the control without IGF-1 or LY294002 treatment at each concentration of CAPE.

Insulin or insulin-like growth factor-1 IGF-I receptor signaling pathway activates phosphoinositide 3-kinase (PI3-kinase) and its downstream target Akt [115–117]. The activation of Akt by IGF-1 treatment also promotes protein synthesis via stimulation of mammalian target of rapamycin (mTOR) and p70 S6 kinase, both are downstream targets of Akt, and thereby inhibiting activity of glycogen synthase kinase 3 (GSK3) [118,119]. Stimulating LNCaP 104-S or PC-3 cells with 200 ng/mL IGF-1, the ligand for IGF-1 receptor and an up-stream agonist primarily for the PI3K-Akt signaling pathway, partially rescued the suppressive effect of CAPE (unpublished data, Figure 5A,C). Treating LNCaP 104-S cells with 200 ng/mL epidermal growth factor (EGF), the ligand for EGFR and a potent agonist for the MAPK and Stat pathways, did not block the suppressive effect of CAPE (Figure 5A). Adding PI3K inhibitor LY294002 with CAPE to LNCaP cells caused only moderate additive growth inhibition compared to CAPE treatment alone (unpublished data, Figure 5B). Co-treatment of CAPE with LY294002 did not induce additive growth inhibition compared to LY294002 treatment alone in PC-3 cells (Figure 5C). Taken together, our results suggest that the PI3K-Akt pathway is a major pathway target for CAPE treatment although CAPE treatment alone is not able to completely block PI3K/Akt signaling in human prostate cancer cells.

## 8. Potential Clinical Application of CAPE

Oral administration of 10 mg/kg CAPE for six weeks caused 50% reduction of tumor growth of LNCaP xenografts while i.p. injection of 10 mg/kg of CAPE for five weeks caused 33% reduction in tumor growth of PC-3 xenografts. The EC_50_ of 96 h CAPE treatment to cause growth inhibition in advanced human prostate cancer cell lines was approximately 0.7 μM to 18.7 μM [70,71]. The achievable concentration of CAPE in human serum is approximately 17 μM [120]. It is therefore possible to use CAPE as an adjuvant therapeutic agent for advanced prostate cancers.

The pharmacokinetic profile of CAPE was determined in rats after intravenous (i.v.) administration of 5, 10 or 20 mg/kg. The plasma concentration of CAPE was measured with liquid chromatography tandem mass spectrometry and was estimated using non compartmental analysis (NCA) and biexponential fit [121]. Total body clearance values for CAPE ranged from 42.1 to 172 mL/min/kg and decreased with the increasing dose of CAPE. The volume of distribution values for CAPE ranged from 1555 to 5209 mL/kg and decreased with increasing dose. The elimination half-life for CAPE ranged from 21.2 to 26.7 min and was independent of dose [121]. This study suggested that CAPE was distributed extensively into animal tissues and was eliminated rapidly with a short half life. Intraperitoneal injection of CAPE at 10–30 mg/kg for 7 days did not affect mice body weight [95]. Plasma alanine amino transferase (ALT) as well as free thiol content and lipid peroxidation in the liver and kidney tissue were measured to study the *in vivo* efficacy and toxicology of CAPE treatment in mice [95]. Seven days of i.p. injection of 10 mg/kg of CAPE showed no toxicity while i.p. injection of higher dose (20 and 30 mg/kg) CAPE caused mild dose-dependent liver and kidney toxicity. Therefore, CAPE treatment of dosage lower than 10 mg/kg is plausible for clinical trial in patients.

CAPE treatments have been shown to sensitize cancer cells to chemotherapeutic drugs and radiation treatment by inhibiting pathways that lead to treatment resistance in animal models [122]. CAPE is a protective agent from therapy-associated toxicities in animal models [122]. Doxorubicin is a chemotherapy drug used for hematological malignancies with side effects including acute renal failure [122]. CAPE treatment protected renal, heart, and brain tissues damages caused by doxorubicin treatment in rats [123–125]. Cisplatin is one of the most widely used chemotherapeutic agents for treatment of solid tumors. CAPE treatment protected liver damage caused by cisplatin treatment [126,127]. Methotrexate is an anti-metabolite and anti-folate drug used in treatment of cancer, autoimmune diseases, ectopic pregnancy, and induction of medical abortions. CAPE treatment protected methotrexate-induced renal oxidative impairment in rats [128]. Bleomycin is a glycopeptide antibiotic generated from bacterium *Streptomyces verticillus*. Bleomycin causes breaks in DNA and is used as a chemotherapy agent for several types of cancers. CAPE treatment inhibited bleomycin-induced lung fibrosis [129]. Tamoxifen is an antagonist of the estrogen receptor (ER) in breast tissue via its active metabolite, hydroxytamoxifen. Tamoxifen is the most common treatment for ER-positive breast cancer in pre-menopausal women. CAPE treatment significantly prevented liver toxicity induced by Tamoxifen treatment [130]. Co-treatment with CAPE and five chemotherapeutic agents commonly used in prostate cancers showed additive suppressive effects in PC-3 cells [71]. CAPE treatment attenuated radiation treatment-induced pulmonary injury *in vivo*[131]. CAPE treatment sensitized colorectal adenocarcinomas to radiation treatment without affecting bone marrow radio-response in animal model [132]. Therefore, treatment with CAPE not only may suppress tumor growth in patients but also may protect patients from chemotherapy or radiation therapy.

## 9. Conclusions

According to the above summaries in this review, there are strong evidences that CAPE treatment suppresses tumor growth and Akt signaling in human prostate cancer cells. CAPE treatment reduces the dosage of chemotherapeutic agents required and protects organ damages and toxicity induced by various kinds of cancer chemotherapy drugs or radiation therapies. Therefore, CAPE is a potential treatment for advanced prostate cancer targeting Akt signaling.

## Figures and Tables

**Table 1:** Characteristics of commonly used human prostate cancer cell lines. Characteristics, including androgen receptor (AR) level, p53 and phosphatase and tensin homolog (PTEN) status, androgen-dependency, and capability to generate prostate-specific antigen (PSA), of LNCaP 104-S, PC-3, and DU-145 cells are listed.

	LNCaP 104-S	PC-3	DU-145
AR	+	−	−
p53	+	−	mutant
PTEN	−	−	+
androgen-dependency	dependent	independent	independent
androgen effect on cell proliferation	stimulation	no response	no response
differentiation	well	poorly	poorly
originality	lymph-node metastases	bone-metastases	brain-metastases
PSA production	+	−	−
EC50 to CAPE treatment (μM)	0.68	18.65	9.54
